# Size and surface modification of silica nanoparticles affect the severity of lung toxicity by modulating endosomal ROS generation in macrophages

**DOI:** 10.1186/s12989-021-00415-0

**Published:** 2021-06-17

**Authors:** Masahide Inoue, Koji Sakamoto, Atsushi Suzuki, Shinya Nakai, Akira Ando, Yukihiko Shiraki, Yoshio Nakahara, Mika Omura, Atsushi Enomoto, Ikuhiko Nakase, Makoto Sawada, Naozumi Hashimoto

**Affiliations:** 1 Department of Respiratory Medicine, Nagoya University Graduate School of Medicine, 65 Tsurumai-cho, Showa-ku, Nagoya, 466-8550 Japan; 2 Graduate School of Science, Osaka Prefecture University, Sakai, Osaka, 599-8570 Japan; 3 Department of Pathology, Nagoya University Graduate School of Medicine, Nagoya, Japan; 4 Department of Brain Function, Research Institute of Environmental Medicine, Nagoya University, Nagoya, Japan; 5 Department of Molecular Pharmacokinetics Graduate School of Medicine, Nagoya University, Nagoya, Japan

**Keywords:** Silica nanoparticle, NADPH oxidase, Endosome, ROS, Macrophage, Lung

## Abstract

**Background:**

As the application of silica nanomaterials continues to expand, increasing chances of its exposure to the human body and potential harm are anticipated. Although the toxicity of silica nanomaterials is assumed to be affected by their physio-chemical properties, including size and surface functionalization, its molecular mechanisms remain unclear. We hypothesized that analysis of intracellular localization of the particles and subsequent intracellular signaling could reveal a novel determinant of inflammatory response against silica particles with different physico-chemical properties.

**Results:**

We employed a murine intratracheal instillation model of amorphous silica nanoparticles (NPs) exposure to compare their in vivo toxicities in the respiratory system. Pristine silica-NPs of 50 nm diameters (50 nm-plain) induced airway-centered lung injury with marked neutrophilic infiltration. By contrast, instillation of pristine silica particles of a larger diameter (3 μm; 3 μm-plain) significantly reduced the severity of lung injury and neutrophilic infiltration, possibly through attenuated induction of neutrophil chemotactic chemokines including MIP2. Ex vivo analysis of alveolar macrophages as well as in vitro assessment using RAW264.7 cells revealed a remarkably lower cellular uptake of 3 μm-plain particles compared with 50 nm-plain, which is assumed to be the underlying mechanism of attenuated immune response. The severity of lung injury and neutrophilic infiltration was also significantly reduced after intratracheal instillation of silica NPs with an amine surface modification (50 nm-NH_2_) when compared with 50 nm-plain. Despite unchanged efficacy in cellular uptake, treatment with 50 nm-NH_2_ induced a significantly attenuated immune response in RAW264.7 cells. Assessment of intracellular redox signaling revealed increased reactive oxygen species (ROS) in endosomal compartments of RAW264.7 cells treated with 50 nm-plain when compared with vehicle-treated control. In contrast, augmentation of endosomal ROS signals in cells treated with 50 nm-NH_2_ was significantly lower. Moreover, selective inhibition of NADPH oxidase 2 (NOX2) was sufficient to inhibit endosomal ROS bursts and induction of chemokine expressions in cells treated with silica NPs, suggesting the central role of endosomal ROS generated by NOX2 in the regulation of the inflammatory response in macrophages that endocytosed silica NPs.

**Conclusions:**

Our murine model suggested that the pulmonary toxicity of silica NPs depended on their physico-chemical properties through distinct mechanisms. Cellular uptake of larger particles by macrophages decreased, while surface amine modification modulated endosomal ROS signaling via NOX2, both of which are assumed to be involved in mitigating immune response in macrophages and resulting lung injury.

**Supplementary Information:**

The online version contains supplementary material available at 10.1186/s12989-021-00415-0.

## Background

Nanomaterials are defined as materials having a one-dimensional diameter of less than 100 nm. Silica nanoparticles have been utilized for various purposes due to their highly adaptable biocompatibility and stability. Application of silica nanomaterials is expected to expand from food and cosmetics to the biomedical industry for functions such as drug delivery and bioimaging [1]. At the same time, increasing chances that the human body will be exposed to them are anticipated. Recent reports have suggested the potential toxicity of silica nanomaterials including nanosilica [2, 3]. Elucidating the mechanisms of toxicity would facilitate biocompatible modification of silica NPs and develop methods of prevention and treatment of potential health hazards.

It is anticipated that toxicities of nanomaterials are mediated by oxidative stress and inflammation [3, 4]. Lysosomal dysfunction or lysosome membrane permeabilization (LMP), which induces the release of toxic effectors (lysosomal iron, cathepsins, cytosolic acidification) and subsequent induction of pro-inflammatory mediators are supposed to be the major underlying mechanisms [5, 6]. Although physico-chemical properties such as size and surface features are known to play an important role in the toxicity of silica NPs [3, 7, 8], the exact mechanisms by which the properties of nanomaterials determine the reaction of cells that uptake them remains to be elucidated. It has long been recognized that reactive oxygen species (ROS) are produced in aerobic cells either as by-products during mitochondrial electron transport or by several oxidoreductases and metal-catalyzed oxidation of metabolites [9]. Stimulated production of ROS was first described in phagocytic cells and named “the respiratory burst”, which is performed by multicomponent nicotinamide adenine dinucleotide phosphate (NADPH) oxidases (NOXs) [10]. The role of the respiratory burst was initially recognized as a bactericidal effect. Since then, NOX-dependent endosomal ROS production has been demonstrated in various cells other than phagocytes, and recognized to serve as an important cellular signal [11, 12]. However, few studies have focused on its involvement in the regulation of immune response in macrophages induced by silica NPs with different properties. In the present study, we compared the in vivo toxic effects of silica nanoparticles with different physico-chemical properties using a murine intratracheal instillation model of fluorescence-labeled amorphous silica nanoparticles. We hypothesized that analysis of intracellular distribution of silica particles and subsequent intracellular signaling might reveal the determinants of inflammatory response against silica particles with different physico-chemical properties. To address this hypothesis, we conducted the lines of in vivo and in vitro experiments.

Our results demonstrated that their size affected the efficiency of the cellular uptake of NPs, while surface modifications with amine moieties altered the proinflammatory reaction of endocytosed particles in macrophages, both of which appeared to contribute to attenuated lung injury. Moreover, in vitro studies suggested that the surface modification of silica-NPs modulates endosomal ROS signaling via NOX2, which leads to the difference in the induction of proinflammatory cytokines including MIP2 in phagocytes.

## Results

### Characterization of the silica particles with different properties

We examined the physicochemical properties of silica particles used in the present study by means of transmission electron microscope, dynamic light scattering (DLS), and FT-IR for assessing their shapes and primary diameters, secondary particle diameters, surface charges in each vehicle, and confirmation of surface modification. The results were summarized in Supplementary Fig. 1, 2, and Supplementary Table 1. We confirmed the spherical shapes and the primary / secondary diameters of two nanoparticles (50 nm-plain and 50 nm-NH_2_) were comparable as evaluated by TEM and DLS. We observed the evidence of amine moiety by FT-IR confirming amine-functionalization on the surface of 50 nm-NH_2_ particles, while their zeta-potentials measured in each vehicle were below zero.

### Intratracheal instillation of silica-NPs with different sizes and surface modifications altered the extent of lung injury

To investigate the relationship of the physico-chemical properties of silica particles to the extent of lung toxicity, we intratracheally instilled amorphous silica particles of different sizes and surface modifications into C57BL/6j mice to induce lung injury. We conducted pilot experiments determining the optimal dosage of intratracheal instillation. The results suggested that low-dose (30 μg) challenges did not clearly show the phenotypic changes enough to distinguish the difference of three tested particles by quantitative assessments of histology as well as chemokine expressions (Supplementary Fig. 3). Therefore, we conducted the following in vivo experiments with a higher dose (400 μg).

**Fig. 1:**
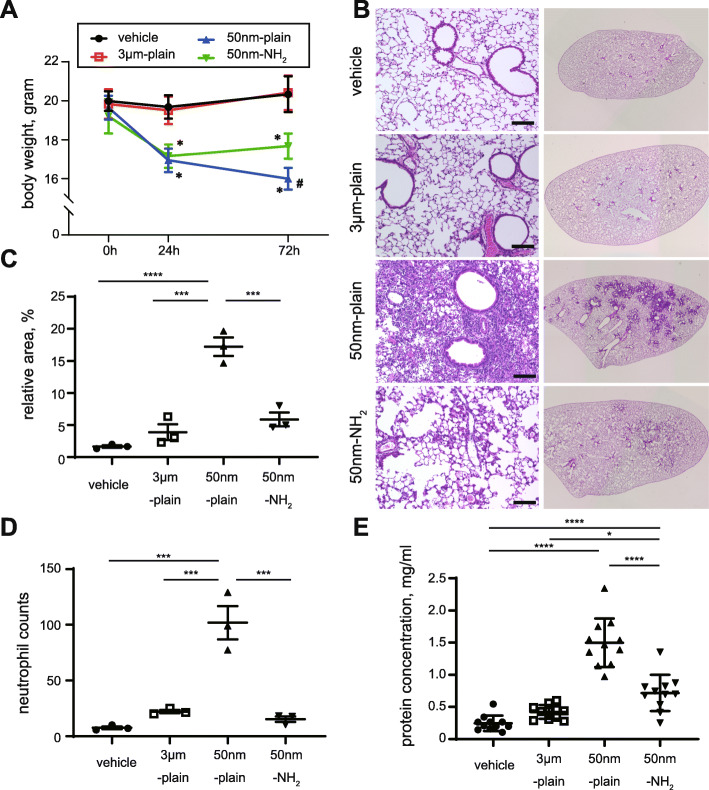
In vivo model of silica-nanoparticle (NP) induced lung injury. **A**. Body weights of mice at different time points (0 h, 24 h,72 h) after intratracheal instillation of silica or vehicle. Data are expressed as mean ± SD. *n* = 11–14 per group. *p < 0.0001 versus vehicle or 3 μm group, #*p* < 0.0001 versus 50 nm-NH_2_ group, respectively by Tukey’s multiple comparison test. **B**. Histological changes in lung tissues 72 h after instillation of silica-NPs were observed by hematoxylin and eosin staining. Left panels show low magnification views and right panels show the appearance of whole left lung lobes. Scale bars = 100 μm. **C**: The lung injury area was quantified by BZ-X Analyzer Hybrid Cell Count Software. An average of three different lobes from each mouse was calculated. Data from at least three mice in each group are shown as mean with 95% CI. *p* = 0.0055. *p < 0.05, **p < 0.01 by one-way ANOVA with Tukey’s multiple comparison test. **D**. Quantitative evaluation of neutrophilic inflammation. Three high power field images including terminal bronchioles of each lung lobe stained with H&E were captured. Infiltrated neutrophils found in each picture were counted and the average number of neutrophils was calculated per mice. Data were summarized as mean ± SEM of three mice. ***p < 0.001, ****p < 0.0001 One-way ANOVA with Tukey’s multiple comparison test. **E**. Total protein concentration in bronchial lavage fluid was assessed by BCA assay. Data are expressed as the mean ± SD. *n* = 11 per group. One-way ANOVA *p* < 0.0001. *p < 0.05, ***p < 0.001, ****p < 0.0001 by Tukey’s multiple comparison test

Mice instilled with silica nanoparticles (NPs) of 50 nm diameter without surface modification (50 nm-plain) exhibited a significant time-dependent reduction in body weight up to 72 h (Fig. 1A). Mice instilled with silica NPs of 50 nm diameter with amine surface modifications (50 nm-NH_2_) exhibited a reduction in body weight comparable to 50 nm-plain at 24 h, but body weight at 24 h remained unchanged up to 72 h. Weights of mice instilled with silica particles of 3 μm diameter without surface modification (3 μm-plain) were not altered compared to that of vehicle-instilled controls. Next, we histologically examined the extent of pulmonary inflammation 72 h after the instillation of silica particles (Fig. 1B). Hematoxylin & eosin (H&E) staining of the tissue samples showed that the lungs of mice instilled with 50 nm-plain NPs exhibited extensive infiltration of inflammatory cells in the air cavities. Meanwhile, the lungs from mice instilled with 50 nm-NH_2_ or 3 μm-plain particles showed less inflammatory cell infiltration. Quantification of inflammation using BZ-X software showed a significant increase in the area filled with dense inflammatory cells in the 50 nm-plain group compared with the control. The 3 μm-plain and 50 nm-NH_2_ groups also showed slight increases in inflammatory cell infiltration compared with the control, albeit not statistically significant (Fig. 1C). Interestingly, we observed a significant increase in the number of neutrophils that infiltrate around the terminal bronchioles in the 50 nm-plain group (Fig. 1D), whereas the lungs of the 3 um-plain group or 50 nm-NH_2_ group showed modest neutrophil infiltration compared to the control.

We further quantified the concentration of total protein in bronchoalveolar lavage fluid (BALF), which reflects the severity of lung injury, obtained from mice instilled with silica-NPs (Fig. 1E) [13]. Compared with vehicle-instilled controls, 50 nm-plain NPs induced a significant increase in the total protein concentration (mean ± s.d. concentration of 1498 ± 379 ng/mL in 50 nm-plain group versus 245 ± 120 ng/mL in vehicle group). On the other hand, BALF from mice instilled with 3 μm-plain exhibited comparable concentrations of total protein to BALF from vehicle-instilled controls (421 ± 108 ng/mL). In addition, the protein concentration of BALF from 50 nm-NH_2_ NP-treated mice was elevated compared to vehicle treated mice (717 ± 282 ng/mL), but significantly attenuated when compared with that of 50-nm plain-treated mice. Taken together, a single intratracheal instillation of nano-size silica NPs induced significant lung inflammation up to 72 h. We also observed that either larger size or amine-surface modification of silica-NPs significantly attenuated the severity of lung inflammation.

### Different properties of silica-NPs affected severities of neutrophilic inflammation and expression of inflammatory chemokines

**Fig. 2:**
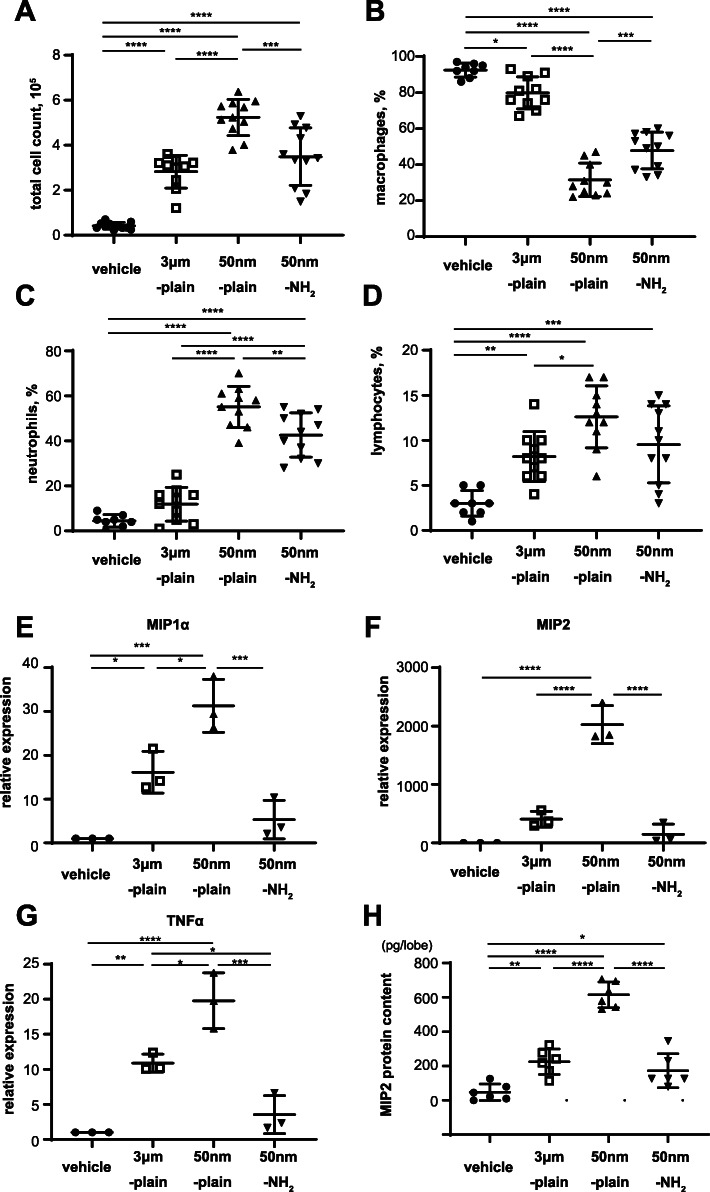
Characteristics of NPs determine inflammatory cell migration and cytokine expressions in murine lung instilled with silica-NPs. **A-D.** Total cell count (**A**) and cell differentials including macrophages (**B**), neutrophils (**C**) and lymphocytes(**D**) in bronchoalveolar lavage fluid 72 h after instillation of silica-NPs. *N* = 11 in each group. **E-G.** Chemokine gene expression in lung tissue 6 h after instillation of silica-NPs. Total RNA was isolated from lung tissue from mice 6 h after silica-NPs instillation, and gene expression of proinflammatory cytokine genes (MIP1α (E), MIP2 (**F**), and TNF-α (**G**)) were determined by qRT-PCR. Data are expressed as mean ± SEM of 3 independent experiments, each comprised of 3 mice per group. **H.** MIP2 protein concentrationsin left lung lobes harvested 6 h after silica particles instillation were determined by ELISA of the lung homogenates. Data represents mean ± SD of six biological replicates. For all the above figures, *p < 0.05, **p < 0.01, ***p < 0.001, and ****p < 0.0001 by ANOVA with Tukey’s multiple comparison tests

We next aimed to characterize the lung inflammation induced by instillation of silica particles with different properties. For this objective, we performed bronchoalveolar lavage at 72 h and analyzed the counts of inflammatory cells in BALF (Fig. 2A-D, and Supplementary Fig. 4). Total cell counts in BALF were significantly increased in mice treated with silica-NPs of all three types, with a remarkable increase in the 50 nm-plain group in comparison with the other two particles (Fig. 2A). We also assessed the percentages (Fig. 2B-D) and absolute numbers (Supplemental Fig. 4) of cell differentials. Percentage and absolute number of neutrophils in BALF were also remarkably increased in mice treated with 50 nm-plain NPs. In mice treated with 50 nm-NH_2_ NPs, BALF neutrophils were still significantly increased but to a lesser extent when compared with mice treated with 50 nm-plain. Meanwhile, the 3um-plain group did not show a significant difference compared to the control group (Fig. 2C and Supplementary Fig. 4B). The increase in lymphocytes was less remarkable compared to neutrophils but showed the similar trends with neutrophils (Fig. 2D and Supplementary Fig. 4C). The percentages of macrophages appeared to be decreased, but the absolute numbers were increased in mice with three particle treatment groups compared with vehicle control group (Supplementary Fig. 4A).

We then sought to characterize the expression of inflammatory chemokines that are responsible for the neutrophilic inflammation induced by silica particles in the lungs. Expression of two chemokines known as potent neutrophil chemo-attractants (MIP1α and MIP2) [14, 15], as well as TNF-α, a proinflammatory cytokine known to be responsible for silica- induced tissue injury and inflammation [16], were assessed (Fig. 2E-G) at 6 h after instillation, which was confirmed by the study determining time-course of chemokine expression (Supplementary Fig. 5). Compared with vehicle-treated controls, significant augmentation in the expression of MIP1α, MIP2, and TNF-α genes was observed in the lungs of the 50 nm-plain group. Expression of MIP1α, MIP2, and TNF-α genes in lungs instilled with 50 nm-NH_2_ NPs and those instilled with 3 μm-plain were modestly increased compared to controls, but significantly attenuated compared to 50 nm-plain group. We also confirmed changes in MIP2 protein level in lung tissues by ELISA (Fig. 2H). Taken together, the differences in the extent of lung injury caused by intratracheal silica particles with different properties were accompanied by different extents of neutrophilic infiltration and chemokine expression.

### Alveolar macrophages predominantly took up silica NPs in lungs instilled with silica-NPs with different properties

**Fig. 3:**
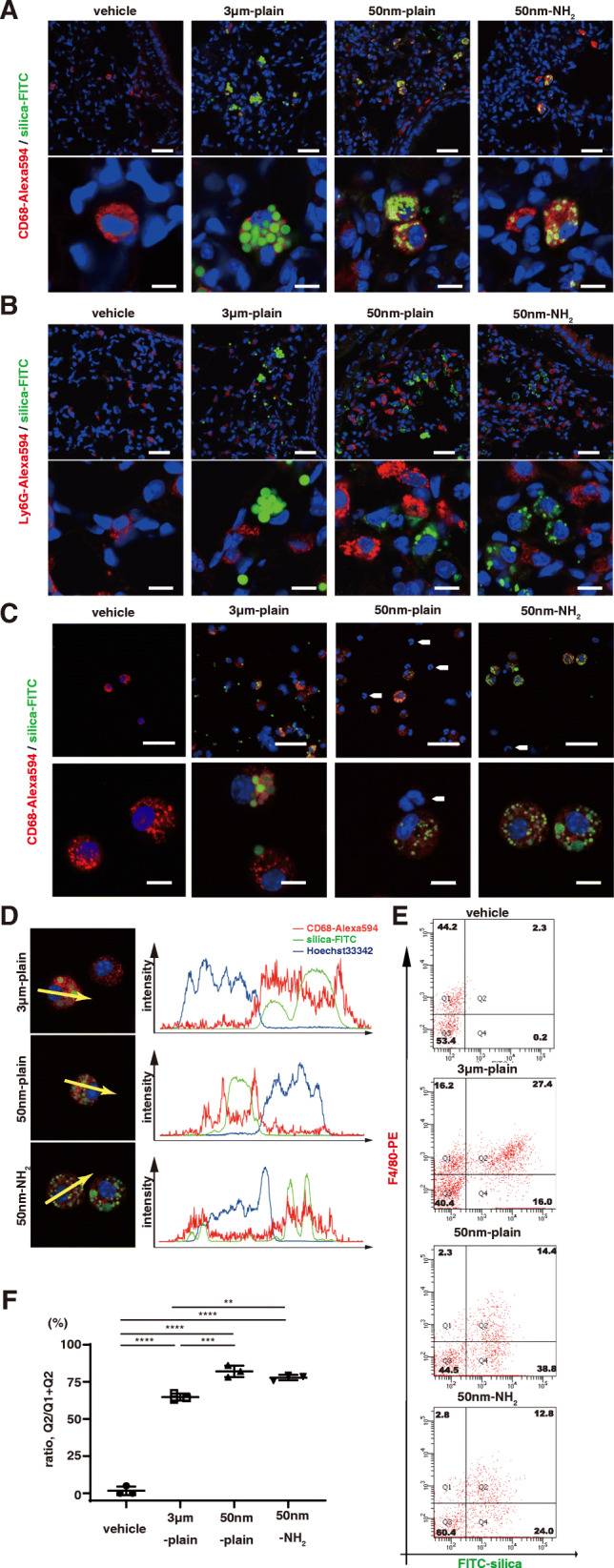
Silica-NPs accumulated in alveolar macrophages of silica-NPs instilled murine lungs. **A-B.** Distribution of silica particles in murine lungs was studied by confocal fluorescence microscopy. **A.** Representative images showing the localization of silica-NPs and macrophages labeled with anti-CD68 antibody. In the images, blue indicates cell nucleus, green indicates FITC-labeled silica particles, and red indicates Alexa 594 labeled CD68. Scale bars in low magnification (top panels) = 50 μm, in high magnification (bottom panels) = 10 μm. **B.** Representative images showing the localization of silica-NPs and neutrophils labeled with anti-Ly-6G antibody. In the images, blue indicates cell nucleus, green indicates FITC- labeled silica particles, and red indicates Alexa 594 labeled Ly6G. The white arrows indicate Ly-6G-postive cells with multi-segmented nucleus, which are absent from FITC-labeled silica-NPs. Scale bars in low magnification (top panels) = 50 μm, in high magnification (bottom panels) = 10 μm. **C-D.** Distribution of silica particles in cells in BALF were studied by confocal fluorescence microscopy. **C.** Representative images showing the localization of silica-NPs and macrophages labeled with anti-CD68 antibody. In the images, blue indicates cell nucleus, green indicates FITC-labeled silica particles, and red indicates Alexa 594-labeled CD68. Note that the CD68-negative, smaller cells with multi-segmented nucleus indicated by white arrows, are absent from FITC-labelled silica-NPs. **D.** Plots of the fluorescence intensity of CD68 (red), FITC-labelled silica-NPs (green), and nucleus (blue) over a cross-section along the indicated yellow arrows. Data are representative of at least three independent experiments. **E-F.** Flow cytometric detection of intracellular silica-NP accumulation in cells retrieved from bronchoalveolar lavage. BAL cells from three animals were pooled in each experiment. **E.** Representative histograms. **F.** The ratio of FITC-positive cells in F4/80-positive macrophages**.** Data are shown as mean ± SD of three independent experiments. **p < 0.01, and ***p < 0.001 by ANOVA with Tukey’s multiple comparison tests

We next sought the localization of silica-NPs in the injured area of the lungs. To this end, we intratracheally instilled FITC-labeled silica particles and examined the lung histology of mice 72 h later by confocal microscopy with immunofluorescent staining. Lung sections were stained with antibodies to anti-CD68 (a macrophage marker) or anti-Ly-6G (a neutrophil marker). In the lungs from all three groups, FITC-labeled particles were predominantly localized within CD68+ mononuclear macrophages (Fig. 3A, and Supplementary Fig. 6,7,8). Meanwhile FITC+ particles were hardly observed within Ly-6G+ neutrophils (Fig. 3B). We also examined the distribution of silica particles in BALF cells by immunofluorescence using an anti-CD68 antibody (Fig. 3C). We observed CD68+ mononuclear macrophages containing FITC-labeled silica particles, while CD68-polymorphonuclear cells (neutrophils) were largely absent from FITC+ silica particles. Line scan analysis of confocal microscopy indicated that signals of FITC-labeled particles distributed on the cells (Fig. 3D).

We then aimed to evaluate the percentage of macrophages that endocytosed silica particles. Flow cytometric analysis of BALF cells with macrophages distinguished by an F4/80 signal revealed that the percentages of FITC-containing macrophages were significantly higher in BALF cells from 50 nm-plain (82.1 ± 3.9%; mean ± SEM) and 50 nm-NH_2_ (77.9 ± 1.9%) silica groups when compared with cells from the 3 μm-plain group (64.7 ± 2.3%) (Fig. 3E,F).

### Different properties of NPs determined the efficacy of endocytosis and expression in chemokines in RAW264.7 cells treated with silica-NPs

**Fig. 4:**
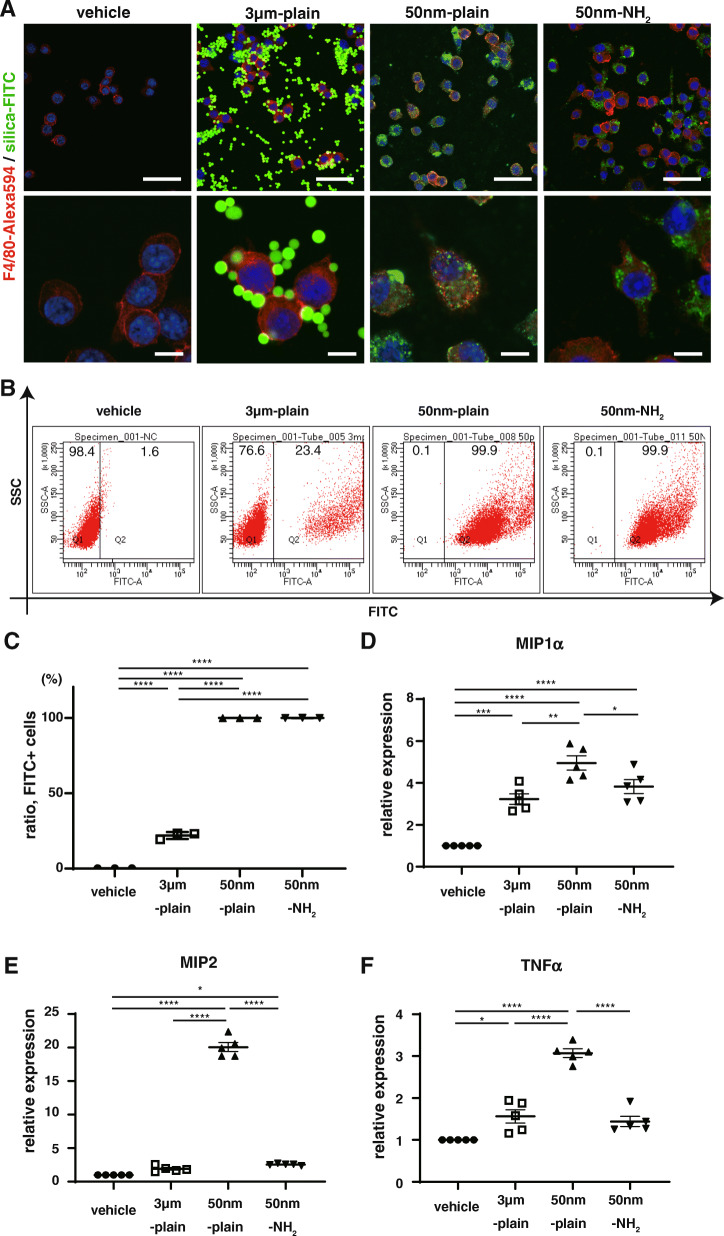
Size and surface modification indicate uptake and subsequent inflammatory response of Raw264.7 cells to silica-NPs. **A.** Cellular uptake of silica particles was studied by immunofluorescence in vitro. Raw264.7 cells, a murine phagocytic cell line, were exposed to different silica-NPs, and cellular uptake was assessed 6 h later by confocal microscopy. In the images, blue indicates cell nucleus, green indicates FITC-labeled silica particles, and red indicates Alexa 594-labeled anti-F4/80 antibody as a cell surface marker of murine macrophages. Scale bars in low magnification (top panels) = 50 μm, and in high magnification (bottom panels) = 10 μm. **B-C.** Flow cytometric detection of intracellular silica-NP accumulation in Raw264.7 cells after 6 h endocytosis. Representative scatter plots showing endocytosis of FITC-labeled silica-NPs are shown in **B.** The graph in **C** shows the proportions of cells that endocytosed FITC-labeled silica particles. **D-F.** Cytokine expressions in RAW264.7 cells 6 h after exposure to silica-NPs. Gene expressions of inflammatory cytokine genes (MIP1α(D), MIP2(E), and TNF-α(F)) were determined by qRT-PCR. Results are shown as mean ± SEM of five independent experiments

Our in vivo experiments revealed that macrophages were the predominant cells that endocytosed silica particles instilled in the lungs. To elucidate the mechanisms by which particles with different properties induced altered levels of lung inflammation, we conducted an in vitro assessment of the response of macrophages to silica particles. Confocal microscopy of murine macrophage cell line RAW264.7 cells incubated 6 h with each silica particles demonstrated that most 50 nm-plain and 50 nm-NH_2_ silica-NPs had been internalized in RAW cells, while a small fraction of 3 μm-plain particles had been endocytosed at the same time point (Fig. 4A, and Supplementary Fig. 9). We confirmed the observed difference in NP uptakes by flow cytometry. RAW cells incubated with 50 nm-plain and those with 50 nm-NH_2_ had > 99% positive FITC signals, while less than 50% of cells incubated with 3 μm -plain were positive for FITC (Fig. 4B-C). Taken together, this suggests that uptake of silica particles of 3 μm diameter by RAW cells was less efficient compared with that of smaller (50 nm diameter) silica-NPs. We also validated these findings in another murine macrophage cell lines, J774.1 and MH-s (Supplementary Figure 10AB).

Next, we assessed the expression of proinflammatory chemokines in RAW cells treated with each silica particles. Treatment with 50 nm-plain significantly increased the gene expressions of MIP1α (Fig. 4D), MIP2 (Fig. 4E), and TNF-α (Fig. 4F). As expected, induced expressions of chemokines in RAW cells treated with 3 μm-plain were significantly lower compared with those in cells treated with 50 nm-plain (Fig. 4D-F), which was assumed to reflect the difference in uptake of particles. Meanwhile, inductions of chemokines in cells treated with 50 nm-NH_2_ were also lower compared with those of cells treated with 50 nm-plain, and were comparable to cells treated with 3 μm-plain (Fig. 4D-F). MIP2 is known to be important chemokine responsible for silica induced lung injury [17], and marked difference in the gene expression observed between groups seemed well correlated with the extent of pathological changes. We also confirmed the changes in secreted MIP2 protein levels in cell culture supernatants of RAW cells treated with each particle by ELISA (Supplementary Fig. 11). Furthermore, difference in MIP2 expression induced by the three silica particles was replicated in J774.1 and MH-s cells (Supplementary Fig. 10C).

### Endocytosed silica-NPs located in endo-lysosomes did not induce massive lysosomal membrane damage

**Fig. 5:**
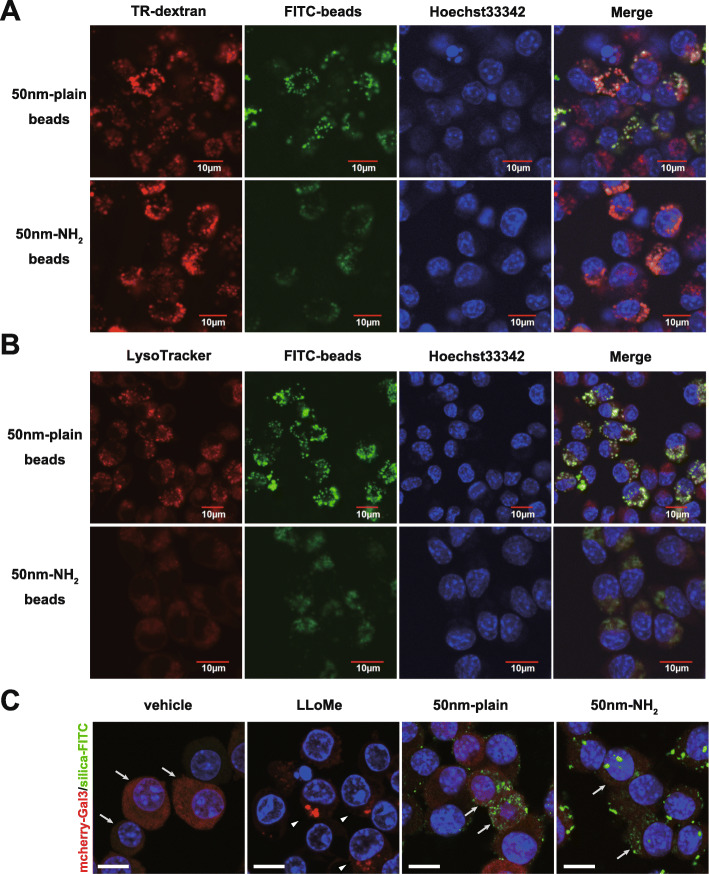
Evaluation of intracellular localization of silica-nanoparticles and endo-lysosomal membrane damage. **A-B.** Images of confocal microscopy showing intracellular distribution of FITC-labeled silica particles. RAW264.7 cells were incubated with indicated silica-NPs for 24 h in the presence of **A.** Texas Red-labeled dextran (TR-dextran; red) or **B.** LysoTracker (red in B) in RAW cells. Cellular nuclei were visualized with Hoechst33342. **C.** Evaluation of lysosomal membrane permeabilization (LMP) in Raw264.7 cells that endocytosed silica-NPs with/without surface modification. Raw264.7 cells transfected with pmCherry-Gal3 (LMP-reporter) were incubated with silica-NPs (50 nm-plain or 50 nm-NH_2_) or LLoMe (LMP inducer) for 6 h. Images were obtained by confocal microscopy. Arrows indicate cells with diffuse cytosolic signal of mCherry-tagged Gal3. Arrowheads indicate cells with punctate signals of accumulated Gal3, suggesting lysosomal membrane rupture. Scale bars in low magnification (top panels) = 50 μm, in high magnification (bottom panels) = 10 μm

Analysis of alveolar macrophages in vivo and RAW264.7 cells in vitro suggested that efficiencies in uptake of 50 nm-plain and 50 nm-NH_2_ NPs were comparable. To explore the mechanisms by which surface modification of silica-NPs changed the chemokine expression of endocytosed cells, we first investigated the intracellular localization of each silica-NP. Confocal microscopy using Dextran (an endosome marker) (Fig. 5A) and LysoTracker (a lysosome marker) (Fig. 5B) demonstrated that most of both silica-NPs co-localized in the endo-lysosomal compartment. (Supplementary Fig. 12) Given that lysosomal membrane permeability (LMP) is one common mechanism of lysosome dysfunction that results in leakage of lysosomal contents and activation of toxic effectors, we hypothesized that silica-NPs with different surface modifications may differently affect the integrity of the lysosomal membrane and cause LMP. To address this hypothesis, we employed an mCherry-galectin-3 system [18] in RAW264.7 cells to visualize LMPs as shown by positive mCherry-Gal3 puncta. Confocal microscopy of mCherry-Gal3-RAW cells treated with both silica-NPs revealed no apparent activation of LMP; meanwhile, mCherry-Gal3-RAW cells treated with a lysosomotropic reagent, LLoMe [19] (Supplementary Fig. 13), exhibited positive Gal3-puncta in the cytosol (Fig. 5C, and Supplementary Fig. 14), suggesting that no obvious LMP developed in the cells that endocytosed 50 nm-plain and 50 nm-NH_2_ NPs.

### Induction of endosomal ROS conferred differential expression in proinflammatory chemokines triggered by silica-NPs of different surface modifications

**Fig. 6:**
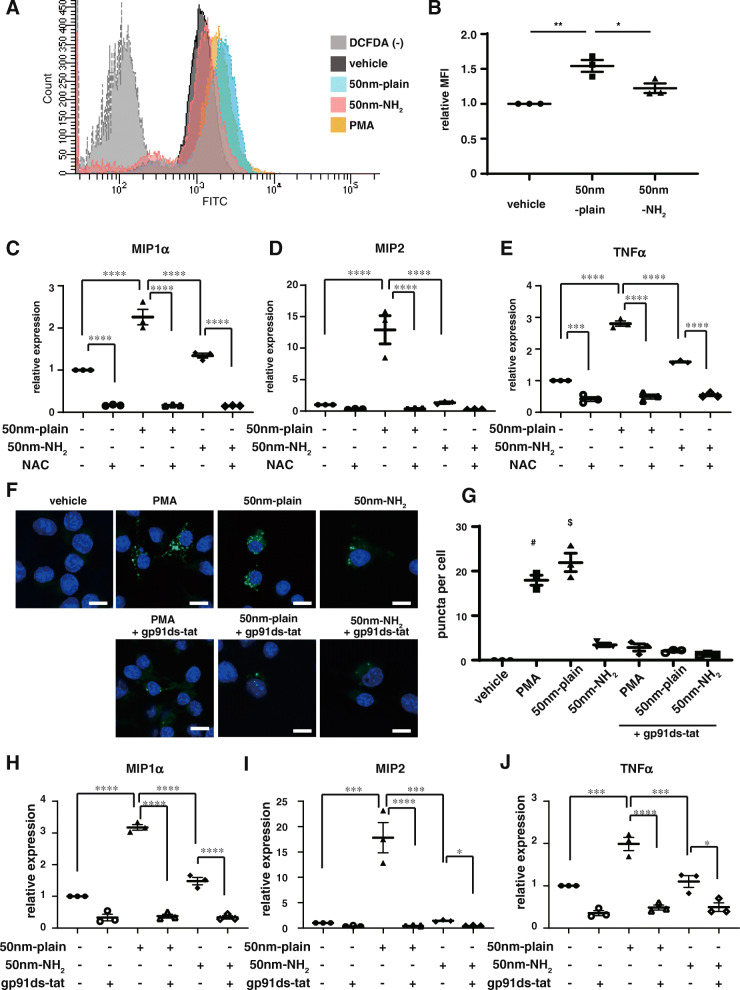
NOX2 inhibition suppressed endosomal ROS generation and chemokine induction by silica NPs in vitro. **A-B.** Intracellular levels of ROS signals in Raw264.7 cells that endocytosed silica-NPs. Raw264.7 cells incubated with silica-NPs for 6 h were stained with DCFDA (intracellular ROS indicator), and signals were quantified by flowcytometry. **A.** Representative histography of fluorescent intensity of RAW cells by DCFDA assay. DCFDA(−) represents vehicle treated cells without staining with DCFDA (negative control). **B.** The graph shows relative levels of intracellular ROS normalized by signals in untreated cells. **C-E.** Chemokine gene expression in RAW264.7 cells incubated with silica-NPs in the presence of N-acetylcysteine (NAC). Total RNA was isolated from RAW264.7 cells 6 h after silica-NPs exposure with or without addition of 10 mM of NAC, and gene expression of proinflammatory cytokine genes (MIP1α (C), MIP2 (E), and TNF-α (F)) was determined by qRT-PCR. **F-G.** Assessment of endosomal ROS levels in RAW264.7 cells with different silica-NPs using OxyBURST. Cells were incubated with silica-NPs (50 nm-plain, 50 nm-NH_2_) for 2 h or PMA for 30 min as a positive control. Some experiments were done with cells pre-treated with gp91ds-tat, a specific inhibitor of NOX2. **F.** Representative images obtained by confocal microscopy. Scale bars = 10 μm **G.** Average number of puncta indicating the signals of endosomal compartment with increased ROS level per cell. (# *p* = 0.0001 vs vehicle, 50 nm-NH_2_, PMA + gp91ds-tat, 50 nm-plain + gp91ds-tat, and 50 nm-NH_2_ + gp91ds-tat groups, respectively by one-way ANOVA with Tukey’s multiple comparison test) **H-J.** Induction of proinflammatory cytokines in Raw264.7 cells treated with silica-NPs was suppressed by treatment with gp91ds-tat, a specific inhibitor of NOX2 activation. For Figs. B, C, D, E, H, I, and J, * *p* < 0.05, **p < 0.01, ***p < 0.001, and ****p < 0.0001 by one-way ANOVA with Tukey’s multiple comparison tests

We next investigated the intracellular ROS levels in RAW264.7 cells treated with 50 nm-plain and 50 nm-NH_2_ NPs by a DCFDA assay (Fig. 6A). Flow cytometric analysis revealed significantly increased intracellular ROS levels in cells treated with 50 nm-plain NPs, while observed intracellular ROS levels in cells treated with 50 nm-NH_2_ NPs were not significantly augmented compared with controls (Fig. 6B). We then aimed to assess the involvement of intracellular ROS in the regulation of proinflammatory chemokines induced by silica-NPs. To this end, we treated RAW cells with silica-NPs in the presence of an antioxidant, N-acetyl cysteine (NAC). As expected, expression of chemokines augmented by treatment with 50 nm-plain NPs was suppressed by co-incubation of RAW cells with NAC to comparable levels with cells treated with 50 nm-NH_2_ NPs (Fig. 6C-E).

ROS is now recognized as a critical component of intracellular signaling. Understanding compartment-specific elements of ROS generation is critical for better dissecting complex ROS-mediated signaling cascades [20]. Specifically, the endosomal ROS generated by the NOXs in phagocytes is critical not only for innate immune defense against certain microbial pathogens, but also for intracellular signaling generated in response to a wide variety of stimuli. We therefore focused on ROS in endosomal components. We utilized OxyBURST Green reagent (H2HFF-BSA) to evaluate endosomal ROS levels in RAW264.7 cells treated with silica-NPs with or without amine-surface modification (Fig. 6F-G). OxyBURST loaded in endosomes can become visible as fluorescent signals once endosomal ROS levels increase. RAW cells preloaded with OxyBURST and stimulated with PMA showed multiple fluorescent dots, indicating endosomes with elevated ROS levels. Interestingly, RAW cells preloaded with OxyBURST and treated with 50 nm-plain NPs exhibited formation of multiple fluorescent dots in the cytosol, while OxyBURST-preloaded cells treated with 50 nm-NH_2_ NPs appeared to show fewer fluorescent dots (Fig. 6F, top panels). We further investigated the involvement of NADPH oxidase 2, a canonical NOX responsible for generation of endosomal ROS in phagocytes. For this purpose, we treated cells with gp91ds-tat, a chimeric peptide with a selective inhibitory effect on NOX2 activation [21]. Addition of gp91ds-tat significantly suppressed the formation of fluorescent dots in cells treated with 50 nm-plain and 50 nm-NH_2_ NPs (Fig. 6F, bottom panels), indicating that NOX2 is responsible for the generation of endosomal ROS induced by endocytosed silica-NPs. Quantification of fluorescent dots of endosomal ROS confirmed that significant differences in the magnitude of endosomal ROS signals were induced by 50 nm-plain and 50 nm-NH2 NPs, both of which were suppressed to levels comparable to the baseline by NOX2 inhibition by gp91ds-tat (Fig. 6G). Finally, we treated RAW cells with silica-NPs and gp91ds-tat together to clarify whether increased endosomal ROS production by NOX2 regulates chemokine expression. As shown in Fig. 6H-J, addition of gp91ds-tat significantly suppressed induction of proinflammatory chemokines MIP1a, MIP2, and TNF-α by silica-NPs, to comparable levels of RAW cells without silica NP stimulation. Likewise, inhibition of NOX2 activation by gp91ds-tat suppressed induction of chemokine expression in J774.1 and MH-s cells (Supplementary Fig. 8D).

## Discussion

In the present study, we evaluated the contributions of size and surface chemistry of silica NPs to the severity of lung toxicity caused by their intratracheal administration. Histopathological assessment of lung injury extent, quantification of alveolar inflammatory cell infiltration in bronchoalveolar lavage fluid, as well as expression analysis of chemokines in the lung tissue, respectively, confirmed the prominent inflammatory cell infiltration predominantly distributed around terminal bronchioles and induced expression of MIP2, a neutrophil chemotactic chemokine in the lungs. Meanwhile, the severity of lung injury with neutrophilic inflammation were attenuated in the lungs treated with silica particles of larger diameter or with amine-surface modification. Tracking fluorescent-labeled silica particles revealed that larger sizes of particles significantly reduced the uptake efficiency of alveolar macrophages, which appeared to be the main type of the cells internalizing silica particles in the lungs. Meanwhile, amine modification of nanosized silica particles did not interfere with the uptake efficiency by phagocytes. We explored the reason of attenuated lung injury by amine-modified silica-NPs in endosomes. Interestingly, significantly lower levels of endosomal ROS were found in RAW264.7 cells that endocytosed amine-modified silica-NPs. Specific inhibition of NADPH oxidase 2, a molecule responsible for endosomal ROS generation in phagocytes [12], abolished the difference in altered chemokine expressions in RAW264.7 cells treated with 50 nm-plain and 50 nm-NH_2_ NPs, suggesting that endosomal ROS generation via NOX2 activation may play a central role in the silica-NP-induced inflammatory reaction in macrophages.

It has been recognized that immune response induced by exposure to engineered nanoparticles such as silica NPs seems greatly affected by their size and shape [3]. The early study by Wottrich [22] showed that smaller silica nanoparticles induced greater inflammatory response in epithelial cells and macrophage cell lines in vivo. Other studies also indicated that smaller particulate size increased the toxicity of silica nanoparticles in epithelial cells [22], endothelial cells [23], and phagocytes [24]. The effect of particulate size on the magnitude of toxicity was further reproduced by in vitro studies. Kaewamatawong et al. assessed histopathological findings of acute (up to 24 h) lung toxicity caused by intratracheal instillation of ultrafine colloidal silica particles (~ 14 nm in diameter) and fine colloidal silica particles [25]. Severer lung toxicity with lung hemorrhage, loss of epithelial cells, and alveolar neutrophil influx was observed in murine lungs instilled with ultrafine colloidal silica particles compared with fine colloidal silica particles. More recently, Kusaka et al. investigated the relationship between the size of amorphous silica particles and inflammatory response [8]. Intratracheal instillation of 30 nm silica particles caused more severe lung inflammation than instillation of 3000 nm silica particles, as assessed by measurement of pro-inflammatory cytokines, neutrophil infiltration in bronchoalveolar lavage fluid, and micro-computed tomography. Our results support these previous findings by assessment of chemokine expression in injured lung tissues, as well as quantitative assessment of histopathologic findings.

Regarding the mechanisms by which the size of silica-NPs determines the levels of inflammatory reaction in phagocytes, Kusaka et al. observed that macrophages internalized silica-NPs irrespective of their diameters [8]. However, the stability of the lysosomal membrane in endocytosed macrophages was differently affected by silica-NPs of different sizes, which they assumed to contribute to the different cytokine inductions including IL-1β. In the present study, the quantitative assessment of FITC-labeled silica NPs led us to speculate that the decreased efficacy in endocytosis may contribute to the diminished inflammatory response in macrophages exposed to the larger (3 μm in diameter) particles. We also examined the instability of the lysosomal membrane of Gal3-mCherry-expressing phagocytes. Exposure to silica-NPs of 50 nm or 3 μm diameters did not cause apparent formation of Gal3 puncta, which is the hallmark of lysosomal membrane permeabilization [26], as shown in the cells treated with LLoMe [19]. Although different experimental conditions (type of cell used and absence of cell priming) might be a cause of the discrepancy observed between the studies, we still assume the difference in cellular uptake between 50 nm-NPs and 3 μm-NPs may be, at least partly, responsible for the different severities of lung injury observed in the in vivo model, because a difference in the efficacy of cellular uptake was also confirmed in the alveolar macrophages obtained in broncholalveolar fluids ex vivo as well as other murine macrophage cell lines (Supplementary Fig. 8B).

Besides their size, it has been shown that the surface modification of silica nanoparticles also plays an important role in determining toxicological effects [27–29]. Amine-modified amorphous silica NPs were shown to be less toxic than unmodified silica NPs in vitro and in vivo [27, 29], although conflicting results have been reported [30, 31]. In the present study, we observed that silica nanoparticles with amine surface modification significantly attenuated the extent of lung injury as well as neutrophilic inflammation when administered intratracheally. This observation supports the recent findings by Morris and colleagues, which showed that intratracheal instillation of bare silica NPs approximately 50 nm in diameter induced more potent inflammation (as assessed by increased neutrophils and protein concentration in BALF) compared to silica NPs with amine surface modification [32]. They further assessed the toxic effects of amine surface modification of silica NPs in an in vitro model using A549 lung epithelial cells, but did not appear to clarify the difference in responses between bare silica NPs and silica NPs with amine surface modification [32]. In the present study, the assessment of localization of fluorescence-labeled NPs demonstrated that most silica NPs instilled in the lungs located inside alveolar macrophages. Accordingly, we examined macrophages, which we assumed to be a main effector in this lung injury model, for the difference in their response to silica NPs. An indispensable role of lung resident macrophages in silica-induced lung injury as well as systemic inflammation was demonstrated previously [33]. Specific depletion of lung macrophages using intratracheal chlodronate dramatically reduced the interalveolar influx of neutrophils as well as the size of venous thromboses (representing systemic inflammatory response). To further assess the difference in responses of phagocytes to silica-NPs with different properties, we employed RAW264.7 cells, a macrophage-like cell line which is often utilized as an in vitro model, to assess reactions of phagocytes to inorganic particles [34, 35]. Our in vitro model well recapitulated the size-dependent efficacy of endocytosis (50 nm versus 3 μm, as mentioned above), as well as the different expressions of chemokines induced by silica-NPs with different properties observed in the murine lungs. A decreased proinflammatory response to amine-modified nanoparticles has been observed using other in vitro models [36]. Taken together, our observation supports the attenuated toxicity of silica NPs with surface modification of the NH_2_- moiety by a lung injury model. It is assumed that observed differences in the severity of lung injury may be attributed to the different responses in alveolar macrophages to endocytosed NPs, including diminished expression of chemokines.

In the process of investigating the mechanisms of attenuated expression of chemokines in macrophages induced by silica-NPs with amine-surface modification, we found a difference in the levels of endosomal ROS induced by the surface modification of silica NPs. While the exact mechanism of silica nanoparticle-induced cellular toxicity has not been elucidated, it is assumed that activation of ROS signaling is involved [37]. Accordingly, we first assessed intracellular ROS levels induced by silica-NPs using DCFDA, and found that amine modification of silica-NPs significantly attenuated intracellular ROS levels in RAW264.7 cells, a result concordant with previous observations [27, 36]. Induction of chemokine expression by silica-NPs was correlated with intracellular ROS levels, and was completely suppressed in the presence with the ROS scavenger NAC, which confirmed that ROS is the central mediator of chemokine induction by silica-NPs. We next asked which compartment inside the cells regulated intracellular ROS signaling. By taking advantage of OxyBURST, which visualizes increased ROS levels inside endosomes [38], we elucidated that silica-NPs without surface modification significantly augmented endosomal ROS levels, while endosomal ROS induced by amine-modified silica-NPs appeared significantly attenuated. We then asked whether induction of endosomal ROS is regulated by a specific molecule, which can be targeted by pharmacological intervention. NADPH oxidases are a family of enzymes that share the capacity to transport electrons across the plasma membrane to generate ROS [12]. NOX2 is predominantly expressed in professional phagocytes such as macrophages and neutrophils. The NOX2 complex is composed of cytosolic components (p47phox, p67phox, p40phox, and the small GTPase Rac1) and the membrane-bound flavocytochrome subunits, gp91phox and p22phox. On stimulation, phosphorylation of p47phox triggers binding of cytosolic components and moves them to the membrane, which results in activation of active NOX2 complex on the membrane. ROS generated by NOX2 plays multiple roles in phagocytes including microbicidal effects when produced in phagosomes and as a second messenger by acting on redox-sensitive signaling pathways [39]. Indeed, previous studies also implicated the involvement of NOXs in the regulation of inflammatory responses against silica-NPs in vivo and in vitro [36, 40]. In the present study, we used gp91ds-tat [20], a chimeric peptide containing a partial sequence of gp91phox that binds to p47phox, for selective inhibition of NOX2. As we expected, selective inhibition of NOX2 by gp91ds-tat was sufficient to suppress augmented endosomal ROS levels and chemokine induction in RAW cells with silica-NPs. Taken together, our results indicate that endosomal ROS signals generated by NOX2 played a central role in the regulation of chemokine induction in the macrophages that endocytosed silica-NPs. It is assumed that the amine surface modification of silica-NPs triggered weaker NOX2 activation and endosomal ROS signaling and attenuated chemokine induction in macrophages, which ultimately resulted in a significantly ameliorated inflammatory response in murine lungs after intratracheal instillation.

While our observation suggested that MIP2 is more responsive against silica particle stimulation compared to MIP1α or TNFα, there are quite large discrepancy between in vivo model and in vitro model. The reason of marked increase of MIP2 in lung tissue may be due to the multiple sources of its expression. A previous study reporting the crystalline silica induced inflammatory response exhibited MIP2 mRNA induction were observed in both macrophages and neutrophils retrieved from BAL [41]. We also supposed that marked increased in MIP2 expression in lung tissue may be the reflection of the summation of stimulated resident cells (epithelium, and macrophage) as well as recruited inflammatory cells (neutrophil and monocyte) from circulation. Even though, induction of MIP2 as well as MIP1 mRNA in alveolar macrophages seem to be important reaction in particle-induced lung inflammation as intratracheal instillation studies with SiO_2_ and TiO_2_ [14] showed that inflammatory doses increase MIP1α and MIP2 mRNA expression preceded the accumulation of inflammatory cells in the lungs.

**Fig. 7:**
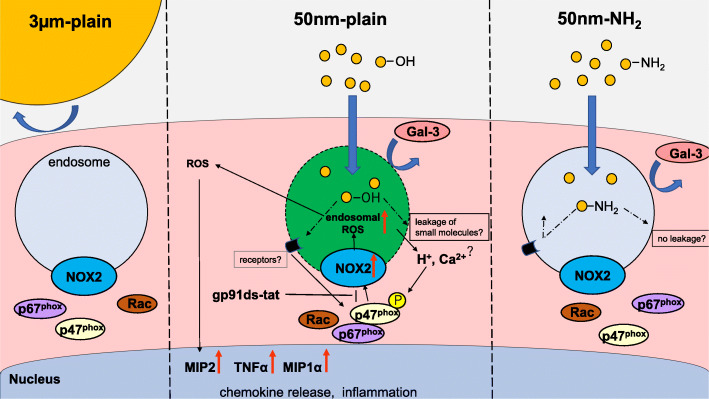
Proposed model for regulation of chemokine expression in macrophages against silica particles with different properties. (Center) Nano-sized pristine silica NPs taken up by macrophages in the lungs were internalized in the endosomes. Interaction between silanol moieties and endosomal membrane does not cause endosomal rupture which can be visualized by Gal3 puncta, but may cause small damage to the membrane, which may lead to calcium and/or proton ion leakage into the cytosol, resulting in assemble of active NOX2 complexes and generation of endosomal ROS signals. Alternatively, certain receptors on the endosomal membrane might specifically recognize pristine silica NPs and activate signaling cascade, resulting in NOX2 activation. Endosomal ROS generated by activated NOX2 is supposed to diffuse into cytoplasm and end up inducing expression in proinflammatory chemokines through redox sensitive inflammatory pathways such as NF-kB. (Left) Efficacy in uptake of larger silica particles (3 μm) by macrophages is significantly reduced compared with nanoparticles, which results in attenuated inflammation. (Right) Nano-sized silica NPs with amine surface modification are internalized in the endosome of macrophages with an efficiency comparable to that of pristine NPs. However, attenuated endosomal ROS signals and inflammatory response were observed This may be because reduced silanol moieties on the surface of NPs may reduce the damage to endosomal membranes or impaired recognition of NH2-coated silica surfaces by the receptors

One of the limitations of the current study is that the mechanism by which surface modification of silica-NPs regulates NOX2 remains undefined. As shown in Fig. 7, we summarized our current findings along with proposed hypothesis. Although we did not observe apparent evidence suggestive of damage to the endolysosomal membrane in RAW264.7 cells with the mcherry-Gal3 system [18], a recent report suggested endocytosis of disruptive materials causes small ruptures of endolysosomes in phagocytes that allow leakage of small molecules such as proton and calcium ions and trigger membrane repair [42]. It is assumed that the density of silanol moieties might affect the severity of endolysosomal small leakage, NOX2 activation, and determine the difference in chemokine induction by silica-NPs with and without surface modification with an amine moiety. Another explanation for the difference in the inflammatory responses is scavenger receptors (SR). SRs such as SR-A, SR-B1, and MARCO are also considered to be molecules responsible for recognition of silica particles and therefore involved in the regulation of subsequent inflammatory responses [37, 43, 44]. The effects of cationic surface modification with an amine moiety may change the affinity of silica-NPs with these receptors and modulate downstream NOX2 activation. Further study to identify the molecular determinants that sense surface modification of silica-NPs is warranted.

Another limitation is that our in vivo model used intratracheal instillation of relatively high dose of silica particles for better discrimination of the phenotypic changes induced by different particles. It is reported that delayed clearance of particles without significant toxicity from alveoli induces inflammation, fibrosis, and especially in rats, even lead to tumorigenesis. This is known as the ‘overload particle hypothesis’ [45]. Rodents such as rats and mice are considered to be more susceptible to neutrophilic alveolar inflammation by particle overload when compared to primates or humans [46]. Thus, it requires extra caution to extrapolate our current findings to the assessment of human health hazard. Nonetheless, because of the technical simplicity and assured dosage given to lower airways, intratracheal instillation has been widely used for studies assessing toxicity of respirable particles. One of the best applications includes comparative toxicity assessment as we did in this study. Therefore, we strongly believe our in vivo study reflects the physiological response to silica particles with different properties to a certain extent.

## Conclusions

In summary, our murine model suggested the dependency of pulmonary toxicity by amorphous silica particles on the physico-chemical properties of particles through distinct mechanisms. Cellular uptake of larger particles by macrophages was decreased compared to smaller particles, while surface amine modification modulated endosomal ROS signaling via NOX2, both of which are assumed to be involved in mitigating the inflammatory response in macrophages and resulting lung injury. Despite the limitation of our in vivo model with intratracheal instillation of high doses of particles, our results indicate that modulation of endosomal ROS signaling via NOX2 might be a target for development of preventive and therapeutic approaches against potential health hazard of silica nanoparticles.

## Methods

### Materials

Amorphous silica nanoparticles (50 nm diameter without surface modification; 50 nm-plain with amine surface modification; 50 nm-NH_2_) and microparticles (3 μm diameter; 3 μm-plain) used in this study were purchased from Micromod Partikeltechnologie GmbH (Rostock, Germany). In some experiments, we used nanoparticles labeled with FITC for their localization. All the products contained no detectable endotoxin as assessed by a ToxinSensor Endotoxin Assay kit (GenScript, Piscataway, USA). Prior to in vitro and in vivo studies, the particles were vortexed for 60 s.

Monoclonal rat anti-CD68 antibody (Clone FA-11) was purchased from Bio-rad (Hercules, CA, USA). Anti-F4/80 antibody (CI: A3–1) was from (Caltag Laboratories, Burlingame, CA, USA). Anti-Ly-6G-antibody (Clone REA526) was from Miltenyi Biotec **(**Bergisch Gladbach, Germany). Hoechst33342 was from Dojindo (Kumamoto, Japan). L-leucyl-L-leucine methyl ester (hydrochloride) was from Cayman Chemical (Ann Arbor, MI, USA). N-acetyl-L-cysteine (NAC) was from Wako Pure Chemicals (Osaka, Japan). Diphenyleneiodonium chloride (DPI) was from Cayman Chemical. gp91ds-tat was from Anaspec (Fremont, CA, USA).

### Murine silica-induced lung injury model

C57BL/6 J female mice (8–10 wks old) were purchased from Jackson Laboratory (Charles River Laboratories Japan, Yokohama, Japan). They were kept in isolated and ventilated cages (3–5 mice per cage) and maintained under standard lab conditions (12-h light/dark cycle) with food and water provided ad libitum. All mouse care and handling protocols were approved by the University Committee on Use and Care of Animals at Nagoya University Graduate School of Medicine. Vehicle (sterile water) alone or each silica particle in the vehicle (400 μg/body or 30 μg/body) were administered intratracheally to mice under anesthesia with inhaled isoflurane. Their body weight was measured at baseline and 24 and 72 h after the administration. Mice were euthanized by overdose of intraperitoneal ketamine–xylazine at the time of endpoint analyses.

### Bronchoalveolar lavage fluid (BALF) collection and cell counts

To collect bronchoalveolar lavage fluid (BALF), the trachea was cannulated, the lungs were lavaged three times with PBS (0.7 ml each time), and ~ 1.5 ml of the instilled fluid was consistently recovered. The samples with insufficient recovery (< 70%) were omitted. Total cell numbers were counted with a standard hemocytometer.

BALF was centrifuged at 3000 rpm for 5 min at 4 °C. After centrifugation, supernatants were collected and stored in − 80 °C until the subsequent analysis, while cell pellets were used to prepare cytospins. Smears of BALF cells were prepared with cytocentrifugation using Cytofuge2 (StatSpin, Norwood, MA, USA) at 1000 rpm for 5 min and then stained with May-Gruenwald Giemsa stain. Cell differentiation was examined by counting at least 100 cells using standard hemocytologic criteria to classify the cells as monocytes/macrophages, neutrophils, or lymphocytes.

### BALF protein concentration

Total protein concentration in BALF were determined using a Bicinchoninic Acid Protein Assay Kit (Sigma-Aldrich, St. Louis, MO, USA) following the manufacturer’s instruction.

### Histological analysis of mouse lung tissues

For histological analysis, lungs were fixed in formalin and embedded in paraffin. Four micrometer section containing the whole lobes of the lungs were stained with H&E. Images containing more than three lung lobes of each mouse were obtained using a BZ-9000 microscope (Keyence, Osaka, Japan) with low and high magnification views. For the quantification of injured lung areas, image processing and digital stitching were performed using BZ-X analyzer (Keyence) as described elsewhere [47, 48]. Briefly, partial lung images were stitched to the image of a whole lung lobe, and the percentage of the whole lobe composed of hypercellular areas associated with infiltration of inflammatory cells was determined using BZ-X analyzer software Three lobes were analyzed per animal, and the average percentage of the three lobes was calculated. For quantitative evaluation of neutrophilic inflammation, three high-power field images including terminal bronchioles were obtained from each slide stained with H&E. The number of infiltrated neutrophils found in each field were counted by the board-certificated pathologist (Y.S.) and the average number of neutrophil counts were calculated. Data were summarized as mean ± SEM of three mice per group.

### Analysis of gene expression analysis in lungs

For the analysis of gene expression in lung tissue of mouse models, lungs were harvested at each time point (6 h, 24 h, 72 h) after instillation of silica particles for RNA isolation. Each experiment was conducted with 3 mice per group, and 3 independent experiments were conducted.

### Cell culture and RNA extraction

The mouse macrophage cell lines RAW 264.7, and MH-s were purchased from American Type Culture Collection (Manassas, VA, USA). Another mouse macrophage cell line J774.1 was from RIKEN BRC (Tsukuba, Japan). Cells were cultured in Dulbecco’s Modified Eagle Medium (Sigma-Aldrich) supplemented with 10% fatal calf serum and 1% Penicillin-Streptomycin (Gibco5,000 U/mL, ThermoFisher Scientific). We conducted the experiments with cell with early passage after thawing the vials. All cultures were incubated at 37 °C in a humidified atmosphere with 5% CO_2_.

The cells (5.0 × 10^5^ cells in 1 ml per well) were seeded on 12-well cell culture plates. Twenty-four hours later cells were stimulated with each silica particle (100 μg/ml). This dose was determined based on the result of preliminary dose-response experiment (Supplementary Fig. 15). In the experiment to assess ROS involvement, an ROS inhibitor (NAC; final concentration 10 mM), or gp91ds-tat (final concentration 20 μM) was added simultaneously with silica particles. Six hours later, the cell-culture dishes were washed with PBS and 1 ml TRIzol (Life Technologies Corp., Carlsbad, CA, USA) was added to isolate total RNA. Isolation of RNA was conducted according to the manufacturer’s protocol, and quantified using NanoDrop ND-1000 (Thermo Fisher Scientific) Only samples with A230/260 above 1.6 were used for subsequent analysis. For gene expression assessments, 3–5 independent experiments were conducted with biological replication.

### Quantitative RT-PCR

Realtime PCR assays were carried out using the GoTaq 1-Step RTqPCR System (Promega, Madison, WI, USA) The sequences of oligonucleotide primers used in this study are summarized in Supplemental Table 1. Relative expression levels of each target were normalized to the 18 s rRNA expression signals. Data were shown as mean ± SEM of repeated experiments.

### MIP2 ELISA

Quantification of MIP2 protein levels were conducted using a commercially available ELISA kit, *following* the manufacturer’s instructions (Mouse CXCL2/MIP-2 DuoSet ELISA, R&D systems, Minneapolis, MN. USA). Cell supernatants were centrifuged to remove the cell debris before measurement. For preparation of lung homogenates, whole left lung lobes harvested 6 h after instillation of silica particles were disrupted with 1 mL of PBS using Multi-Beads Shocker (Yasui Kikai, Osaka, Japan), and debris was removed by centrifugation [49]. The supernatant was used for ELISA analysis.

### Flow cytometry

For the analysis of alveolar macrophages in BALF by flow cytometry, cells from 3 mice per group were pooled and stained as follows. Fc receptors (FcRs) were blocked with FcR Blocking Reagent (#130–092-575, Myltenyi Biotic, Bergisch Gladbach, Germany) before staining. Alveolar macrophages then were stained with CD68-biotin antibody (1:100) and streptavidin-Alexa Fluor 594 conjugate. For analysis of the Raw 264.7 cell experiment in vitro, after incubation with silica NPs, Raw 264.7cells were trypsinized and harvested for FACS analysis. Cells then were analyzed using an FACS Canto II flow cytometer (Becton-Dickinson Japan, Tokyo, Japan). A total of 10,000 events were acquired for each analysis. Dead cells and silica particles were gated out depending on forward scatter and side scatter. The percentage of the fluorescent cells relative to the control was taken into account. We conducted three independent experiments.

### Assessment of intracellular ROS level

Levels of intracellular ROS in RAW 264.7 cells were determined using a DCFDA/H2DCFDA-Cellular ROS Assay Kit (ab113851, Abcam, Cambridge, UK) according to the manufacturer’s protocol. Briefly, Raw 264.7 macrophages (5.0 × 10^5^ cells in 1 ml per well) were seeded on 12-well plates and stimulated with silica particles (100 μg/ml) 24 h later. After 3 h stimulation, cells were incubated with 20 μM DCFDA for 30 min. Cells were then trypsinized and washed once with ice-cold PBS. An FACS Canto II flow cytometer were used to determine intracellular ROS levels by DCF fluorescence. In each experiment, the samples were biologically triplicated. The results are shown as mean ± SEM of 3 independent experiments. In the experiments assessing cellular and endosomal ROS, we employed silica particles without FITC labeling in order to measure fluorescent signals. We confirmed that FITC labeling of particles did not induce significant differences in cellular response by induction of chemokine expressions (Supplementary Fig. 16).

### Immunofluorescent studies for cell surface markers

Lung and cell immunostaining were conducted as previously described [50]. For lung tissue, frozen sections were incubated with primary antibodies against CD68 (1:300 dilution) or Ly-6G (1:100 dilution) at 4 °C overnight. For BAL cells, slides with cytospins were incubated with primary antibodies against CD68 (1:300 dilution) after fixation and permeabilized with 0.2% Triton X-100. For RAW 264.7 cells immunostaining, cells were grown in chamber slides with the density of 8.8 × 10^4^ /well and stimulated with silica particles (100 μg/ml). Six hours later, cells were briefly washed with PBS and fixed in 4% paraformaldehyde for 15 min. The slides were then incubated with the primary antibody against F4/80 (1:500 dilution) at 4 °C overnight. In each experiment the slides were then washed with PBS, and reacted with an Alexa Fluor 594-conjugated secondary antibody at room temperature. After nuclear staining with Hoechst 33342, the slides were mounted and scanned by confocal laser scanning microscopy (TiEA1R; Nikon Instech Co., Tokyo, Japan). For localization of FITC signals from silica nanoparticles, imaging software (NIS-Elements AR; Nikon Instech Co.,) was utilized to analyze the fluorescence intensities of silica nanoparticles, F4/80, CD68, and the nucleus. The representative images from at least two independent experiments with biologically duplicated were shown in the result.

### Immunofluorescent studies of intracellular distribution of silica nanoparticles

Assessment of intracellular localization of silica nanoparticles in RAW264.7 cells was conducted as follows. Briefly, the RAW264.7 cells (4.7 × 10^4^ cells) were cultured on 8-well chamber plates (μ-Plate, ibidi GmbH, Grafelfing, Germany) for 24 h, and the cells were treated with silicas nanoparticles (5 μg/ml) for 24 h in the presence of Texas Red-dextran (Life Technologies) (70 kDa, final concentration 0.5 mg/ml) as an endosomal marker. In addition, the cells were treated with LysoTracker-Red (Life Technologies) (final concentration 500 nM) as a lysosomal marker and Hoechst 33342 (80 nM) (Life Technologies) for 15 min prior to microscopic observation. Images of the culture slides were captured using an FV1200 confocal laser scanning microscope (Olympus, Tokyo, Japan) for visualization of colocalized signals of FITC-labeled silica NPs, organelle markers, and nuclei counterstained with Hoechst33342. Representative images from at least two independent experiments with biologically duplicated are shown in the result.

### Raw264.7 cells with LMP reporter

The pmCherry-Gal3 construct was a gift from Prof. Hemmo Meyer (Addgene plasmid # 85662; http://n2t.net/addgene:85662; RRID: Addgene_85,662). Raw264.7 cells (2.0 × 10^6^) were transfected with 2 μg of pmCherry-Gal3 plasmid DNA using Nucleofector2b (Kit V, protocol D-032; Lonza) and seeded on 6-well plates. Twenty-four hours after transfection, cells expressing Gal3 with an N-terminal mCherry-tag were selectively grown in complete medium supplemented with 1000 μg/mL of G418 (Life Technologies) for 5 d. Monoclonal cell lines were further obtained by limited dilution in 96-well plates. Only clones with positive Gal3 puncta signals with LLoMe stimulation observed by fluorescent microscopy were used for the subsequent study. Cells stimulated either with LLoMe (2 mM) or with silica particles were fixed with 4% formaldehyde after 6 h and images were captured by fluorescent microscopy. The representative images from at least two independent experiments with biologically duplicated were shown in the result.

### Evaluation of endosomal ROS signal

Intra-endosomal ROS production was detected using OxyBURST H_2_HFF Green BSA (Thermo Fisher Scientific) as described previously [51, 52]. Briefly, Raw264.7 cells were incubated in the presence of 100 μg/ml OxyBURST H_2_HFF Green BSA for 1 h at 37 °C and then stimulated by the addition of phorbol myristate acetate (PMA) (300 nM) or each silica particle (100 μg/ml, no FITC label). For the assessment of NOX2 inhibition, gp91ds-tat (5 μM) was added simultaneously with PMA or silica particles. Cells were briefly washed with PBS and then fixed in 4% paraformaldehyde for 15 min and evaluated by confocal laser scanning microscopy (TiEA1R). For quantification of the endosomal ROS signal, the green puncta observed by confocal laser scanning microscopy in each cell were counted, and the average number of puncta per cell was calculated by assessing 20 cells per experiment. Experiments were performed three times, and data were expressed as mean ± SEM.

### Statistical analysis

For comparison of data from more than two groups, one-way ANOVA was employed, and the significance of the difference among the groups were tested by Tukey’s multiple comparison test. The Brown-Forsythe test was used to test the distribution equality of the sample and the Shapiro-Wilk test was used to validate the normality. When appropriate, a non-parametric Kruskal-Wallis test was used. In case of time-dependent weight change analysis, two-way ANOVA was employed. SPSS statistics software (Ver.25) (IBM, Chicago, IL, USA) and GraphPad Prism Ver.8 (GraphPad Software, San Diego, CA, USA) were used to conduct statistical analyses and draw graphs. *P*-values < 0.05 were considered statistically significant.

## Supplementary Information

**Additional file 1.** Supplementary Figures 1-16, Supplementary Table 1, and Supplementary methods.

**Additional file 2.** Primer sequences used in the study.

## Data Availability

The datasets used and/or analyzed during this study are available from the corresponding authors on reasonable request.

## Abbreviations

BAL: Bronchoalveolar lavage
BALF: Bronchoalveolar lavage fluid
LMP: Lysosomal membrane permeability
NAC: N-acetyl cysteine
NADPH: Nicotinamide adenine dinucleotide phosphate
NOX2: NADPH oxidase 2
NPs: Nanoparticles
PMA: Phorbol-myristate-acetate
ROS: Reactive oxygen species
