# Supplementary material for: Size and surface modification of silica nanoparticles affect the severity of lung toxicity by modulating endosomal ROS generation in macrophages
Source: Part Fibre Toxicol. 2021 Jun 17;18:21. doi: 10.1186/s12989-021-00415-0 (PMC8210371; doi:10.1186/s12989-021-00415-0)
Supplement: Supplementary file 1 — Additional file 1. Supplementary Figures 1-16, Supplementary Table 1, and Supplementary methods. [file 12989_2021_415_MOESM1_ESM.docx]

**Size and surface modification of silica nanoparticles affect the severity of lung toxicity by modulating endosomal ROS generation in macrophages**

Masahide Inoue^1*^, Koji Sakamoto^1*^, Atsushi Suzuki^1^, Shinya Nakai^2^, Yasunori Shiraki^3^, Akira Ando^1^, Yoshio Nakahara^1^, Mika Omura^2^, Atsushi Enomoto^3^, Ikuhiko Nakase^2^, Makoto Sawada^4^, and Naozumi Hashimoto^1^

Supplementary file 1 containing the following:

Supplementary figures 1–16

Supplementary table 1

Supplementary methods

Supplementary Figure 1

**Transmission electron microscopy images of silica particles used in the study.**

The scale bars are 10 μm for 3μm-plain beads, and 100nm for 50nm-plain and -NH_2_ beads, respectively.

Supplementary Figure 2

**FT-IR spectra of 50nm-silica particles with and without amine-surface modification.**

The red circle indicates the new absorption peaks around 1500 cm^-1^ representing the N–H bending vibration of amine groups in 50nm-NH_2_ silica particles. The peaks for SiO_2_ were observed around 800, 960, and 1100cm^-1^ due to Si–O–Si symmetric stretching, Si–OH stretching, and Si–O–Si asymmetric stretching vibrations, respectively.

Supplementary Figure 3

**Assessment of intratracheal challenge with low dose (30** μ**g) silica particles and comparison with high dose models.**

A. Comparison of body weight changes after intratracheal instillation of silica or vehicle. Absolute changes in body weight of each mouse from baseline were calculated at 24 and 72 h after instillation. Data are expressed as the mean ± SD. n = 3 for low dose groups, n = 11–14 for high dose groups and vehicle controls.

B. Histological changes in lung tissues 72 h after instillation of low dose (30 μg) silica-NPs were observed by hematoxylin and eosin staining. Top panels show low magnification views and bottom panels show high magnification views. Scale bars = 100 μm.

C. Counts of infiltrated neutrophils observed around the terminal bronchioles. n=3. Difference was not significant by one-way ANOVA.

D-F. Chemokine gene expression in lung tissue 6 h after instillation of low dose silica-NPs, and comparison with high dose (400 μg) models. Data are expressed as mean ± SEM of 3 independent experiments consisting of 3 mice in each group. In the comparison between low dose groups, neither MIP1α(D), MIP2(E), or TNFα(F) reached statistical significance by one-way ANOVA.

Supplementary Figure 4

**Absolute numbers of cell types in BAL cells**

(A) Macrophages (B) neutrophils, and (C) lymphocytes in bronchoalveolar lavage fluid 72 h after instillation of silica-NPs. N=11 in each group. *p<0.05, ***p<0.001, ****p<0.0001 by one-way ANOVA with Tukey’s test.

Supplementary Figure 5

**Time course assessment of chemokine expressions in intratracheal lung injury models.**

Gene expressions of MIP2, MIP1α, and TNFα (normalized to 18sRNA) in lungs harvested at the indicated times after intratracheal challenge with silica particles (400 μg/body) were examined, and values are relative to those in the lungs of vehicle-treated isotime controls. Data were expressed as mean ± SEM of three repeated experiments.

Supplementary Figure 6

**Line scan images assessing the intracellular localization of silica NPs in alveolar macrophages.** Distribution of silica particles in murine lungs was studied by confocal fluorescence microscopy, as shown in Figure 3A. Representative images showing the localization of silica-NPs and macrophages labelled with anti-CD68 antibody. Plots of the fluorescence intensity of CD68 (red), FITC-labelled silica-NPs (green), and nucleus (blue) over a cross-section along the indicated yellow arrows. Data are representative of at least three independent experiments.

Supplementary Figure 7

**Unmerged images for immunofluorescent study of lung tissue shown in Figure 3A.**

Scale bars = 10 μm.

Supplementary Figure 8

**Localization of** **3um-plain particles in lung tissue.**

A: Left, low magnification view of immunofluorescent study shown in Figure 3A. Red: CD68-Alexa549, Green: FITC-silica particles, Blue: Hoechst33432. Close-up of the area marked by the white rectangle is shown in the right, overlaid with a phase contrast image. B: Low magnification view of lung tissue instilled with 3um-plain particles, stained with H&E. Close-up images of the areas in black rectangles are shown in the bottom.

Supplementary Figure 9

**Unmerged images for immunofluorescent study of RAW267.4 cells shown in Figure 4A.**

Scale bars = 10 μm.

Supplementary Figure 10

**Size and surface modification indicate uptake and subsequent inflammatory response to silica particles in murine macrophage cell lines, J774.1, and MH-s cells.**

**A-B.** Flow cytometric detection of intracellular silica-NP accumulation in J774.1 cells or in MHs cells after 6 h endocytosis. Representative scatter plots showing endocytosis of FITC-labelled silica-NPs are shown in **A.** The graphs in **B** show the proportions of cells that endocytosed FITC-labeled silica. **C.** MIP2 expressions in J774.1 cells (left) and MH-s cells (right) 6 h after exposure to silica-NPs. Gene expressions were determined by qRT-PCR. Results are shown as mean ± SEM of three independent experiments (n=3 in each group in each experiment). **D.** Induction of MIP2 expression in J774.1(left) and MH-s (right) cells treated with silica-NPs was suppressed by treatment with gp91ds-tat, a specific inhibitor of NOX2 activation. For Figures B, C, and D, * p<0.05, **p<0.01, ***p<0.001, and ****p<0.0001 by one-way ANOVA with Tukey’s multiple comparison tests.

Supplementary Figure 11

**MIP2 protein concentration in the cell culture supernatant of RAW cells treated with silica particles.**

MIP2 protein levels in cell culture supernatants of Raw264.7 cells 6 h after silica particle stimulation were determined by ELISA. Data represents mean ± SD of five biological replicates. p<0.0001 by one-ANOVA. ****p<0.0001 by Tukey’s multiple comparison test.

Supplementary Figure 12

**Colocalization of dextran or LysoTracker with silica beads in the cells after cellular uptake.**

(A, B) Fluorescence intensity along the white lines in merged pictures of TR-dextran (A) or LysoTracker (B) with 50 nm-plain or 50 nm-NH_2_ silica beads taken up by the Raw264.7 cells in Figure 5A, B. Red: TR-dextran (A) and LysoTracker (B), green: each silica bead.

Supplementary Figure 13

**Destabilization of lysosomal functionality by LLOMe treatment.**

Raw264.7 cells were treated with or without LLOMe (2 mM) for 15 min at 37°C prior to cellular staining with LysoTracker and Hoechst33342 for 15 min at 37°C and observation using a confocal laser microscopy. Red signals: LysoTracker, blue signals: Hoechst33342. Scale bar: 50 μm

Supplementary Figure 14

**Unmerged confocal microscopy images of RAW267.4-pmCherry-Gal3 cells shown in Figure 5C.**

Scale bars = 10 μm.

Supplementary Figure 15

**Dose responses of Raw264.7 exposed to silica particles with different properties.**

Raw264.7 cells were stimulated with indicated concentrations of silica particles for 6 h and expressions of MIP2 gene were assessed by qRT-PCR. (n =1 in each condition)

Supplementary Figure 16

**Chemokine expressions in Raw264.7 cells treated with silica nanoparticles with or without FITC labelling.**

*p<0.05, and ***p<0.001 by ANOVA with Tukey’s multiple comparison tests.

*ns* stands for ‘no significant difference’ between cells treated with silica-NPs with same surface modification but with or without FITC labeling.

Supplementary Table 1

**Table 1. Primary particle diameter, hydrodynamic sizes, and ζ Potentials of amorphous silica particles in vehicle solutions**

|  |  | in sterile water | | in DMEM | |
| --- | --- | --- | --- | --- | --- |
| Particles | primary diameter (TEM)  (nm) | secondary particle diameter (nm) | zeta-potential (mV) | secondary particle diameter (nm) | zeta-potential (mV) |
| 3μm-plain | 4030±770 | 4333.33±263.52 | -31.06±0.25 | 4466±194.67 | -3.67±0.62 |
| 50nm-plain | 40.86±6.64 | 45.98±0.31 | -10.09±0.75 | 66.48±0.69 | -9.38±1.72 |
| 50nm-NH2 | 36.40±8.61 | 50.82±0.23 | -30.5±0.61 | 101.5±0.56 | -5.72±0.06 |

Primary diameters of each particle were determined using TEM (JEM-1400Flash, JEOL Ltd., Tokyo, Japan). The diameters of 100 particles were measured and expressed as mean ± standard deviation. The hydrodynamic sizes, and ζ potentials of particles were determined by Zetasizer nano NS (Malvern Instruments, UK)

**Supplementary methods**

**TEM.** Morphology of particles were assessed by transmission electron microscopy (JEM-1400Flash, JEOL Ltd., Tokyo, Japan) The diameters of 100 particles were measured and expressed as mean ± standard deviation.

**Dynamic Light Scattering.** The hydrodynamic sizes, and ζ potentials of particles resuspended in sterile water (as *in vivo* vehicle) and DMEM (as *in vitro* vehicle) were determined by Zetasizer Nano NS (Malvern Instruments, UK).

**FT-IR.** Fourier transform infrared (FT-IR) spectra were recorded on a JASCO FT/IR-4100 spectrometer by KBr method. FT-IR spectra were recorded in the wave number range of 400–4000 cm^-1^ at a resolution of 2 cm^-1^.
