# Supplementary material for: Size and surface modification of silica nanoparticles affect the severity of lung toxicity by modulating endosomal ROS generation in macrophages
Source: Part Fibre Toxicol. 2021 Jun 17;18:21. doi: 10.1186/s12989-021-00415-0 (PMC8210371; doi:10.1186/s12989-021-00415-0)
Supplement: Supplementary file 2 — Additional file 2. Primer sequences used in the study. [file 12989_2021_415_MOESM2_ESM.docx]

| 18s | Se | 5’-ATCACCATTATGCAGAATCCACG-3’ |
| --- | --- | --- |
|  | As | 5’-GACCTGGCTGTATTTTCCATCC-3’ |
| MIP2 | Se | 5’-AGACAGAAGTCATAGCCACTCTCAAG-3’ |
|  | As | 5’-CCTCCTTTCCAGGTCAGTTAGC-3’ |
| TNFα | Se | 5’-CCACCATCAAGGACTCAA -3’ |
|  | As | 5’-CAGGGAAGAATCTGGAAAGG -3’ |
| MIP1α | Se | 5’-TTCTCTGTACCATGACACTCTGC -3’ |
|  | As | 5’-CGTGGAATCTTCCGGCTGTAG -3’ |

Supplementary Table 1: Primer sequences for qRT-PCR
